# Digestion

**Published:** 1869-01

**Authors:** John W. Holt


### AMERICAN JOURNAL OF DENTAL SCIENCE.

Vol. 11. THIRD SERIES--JANUARY, 1868. No. 9.

### ARTICLE I.

### *Digestion.*

By John W. Holt, D.D.S.

By the digestive process the food taken into the alimentary canal for the nutrition of the animal organism, is reduced to a form in which it can be absorbed from the intestine and pass into the circulation, there to undergo further changes in the nutritive process.

The digestive function is peculiar to animals. They require not only the fluids and gases upon which vegetables depend for their support, but a large proportion of substances of organic origin, often in a solid state and insoluble in water, which requires liquefaction before they can be absorbed, or their nutritious elements be eliminated. This process however, is not one of simple chemical solution, but a catalytic transformation of the aliment, by the organic matters of the digestive fluids.

In all animals the general characters of digestion are the same, the essential requirements being a canal through the body, in which the food is subjected to the solvent and cattalytic action of certain fluids. The digestive apparatus and its actions, however, are variously modified in different species of animals, and in all cases wonderfully adapted to their habits and the nature of their food. For instance, in some of the low forms of animal life, the apparatus appears quite simple, consisting of a short, straight canal through the body, guarded at its ends, or even a simple pouch, with walls secreting a fluid possessing the property to digest the little creatures' peculiar food, and perhaps nothing else so well. On the other hand, we find the apparatus externally complicated in the ruminating animals, for their food is often hard and rough, and the nutritious matter in trifling proportion to the bulk of indigestible substances with which it is entangled. The alimentary canal is long and tortuous, and has four distinct stomachs through which the food passes. These organs vary in size, and in their membranous, glandular and muscular structure, in adaptation to the progressive stages of digestion. In human species, however, digestion is much less complicated than in the herbivora ; the atificial preparation of the food, compensated for the comparative simplicity of the apparatus, which closely resembles that of carnivorous animals.

Digestion is commenced in the mouth with mastication, which is an indespensible preliminary to the ready and perfect liquefaction of the food by the digestive fluids, for by the minute subdivision of a body, and the increase of its surface, it is well kown that the susceptibility of solution, or other chemical action, is greatly increased. In the mouth we have, we may say, a perfect apparatus for the mechanical division of the food. Like other parts of the digestive apparatus, however, we find the organs of mastication, including the teeth, tongue, lips, jaws, &c., varied to the food and habits of the animal. In some animals that bolt their food whole, and even *alive*, frequently, the teeth are sharp and thorn like, for seizing and sustaining their prey, and may be considered as simply prehensile. Some animals have strong teeth firmly fixed in powerful jaws, for crushing their prey, cutting and tearing flesh &c., and in others they are broad, strong and rough, for grinding hard substances by lateral motion of the jaws. In man the organs of mastication are well adapted to the mixed diet, cooked and otherwise atificially prepared, and their functions are performed thoroughly. In the process of mastication the tongue, lips and cheeks, are active in turning the bolus and placing the coarsest parts between the teeth at every motion of the jaws, so that with the cutting of the *incisors*, and the crushing and grinding of the *molars*, the food is thoroughly comminuted.

Accessory to the process of mastication, the food is softened and lubricated by the saliva, a fluid of well known appearance, secreted by three pairs of glands, the parotid, submaxillary and sublingual, and the mucous glands and follicles of the mouth. The saliva as found in the mouth, is a mixture from these several sources, the greater part being furnished by the parotid gland. It is a slightly viscid, alkaline fluid, with a specific gravity of about 1005. About .995 of its composition is water, the remaining .005 being made up, principally, of phosphate of lime, soda and magnesia, chloride of sodium and potassium, some waste epithelial matter, and a peculiar organic substance known as *ptyaline*. This latter ingredient, separate as well as in the saliva, possesses the catalytic power to convert boiled starch into sugar, which was once thought to be its designed physiological action, but now it is believed that the function of saliva is altogether physical, it being absolutely necessary to moisten and lubricate the food for mastication and deglution, and to prepare the mouth for all of its functions. It may however, exercise some unknown but important influence in the subsequent process of digestion. Perhaps by affinity it assists the other digestive fluids to penetrate the semi-solid masses of food.

Prof. Dalton estimates the quantity of saliva secreted by a man in full health, at nearly three pounds, avoirdupois, daily. It flows most abundantly during the mastication of agreeable acid and sweet substances. It is almost entirely suspended during sleep.

After the food is reduced to a soft pasty mass by mastication and insalivation, it is carried backward by the tongue and passed through the isthmus of the fauces, into the pharynx, which is drawn up and dilated to receive the bolus. Then, involuntarily the constrictor muscles contract on it, successively, from above downward, and it is forced through the pharynx into the œsophagus, when the muscular action continues, and the food is carried into the stomach. This constitutes the highly complicated function of deglutition.

Next, we have to consider the function of the stomach, the most dilated part of the alimentary canal and the principal organ of digestion.

The active agent in stomach digestion, is the *gastric juice*, a clear, amber tinted, watery fluid, secreted by the glandular follicles which occupy the entire thickness of the mucous membrane. The peculiarities of the gastric juice and stomach digestion, have been studied by physiologists with very decisive results, by means of gastric fistulæ, through which the progress of digestion has been seen, and the fluid obtained for experiment and analysis. From such experiments and observations, much has been learned in regard to the composition of the gastric fluid, the quantity secreted, its action on different kinds of food, the time required for digestion, &c.

The chemical reaction of the gastric juice is distinctly acid; but in regard to the acid giving the reaction, opinions are various. The analyses by some physiologists, give *lactic acid;* others have found *hydrochloric*, and otheos still believe that both may be present. They generally agree, however, that there is a free acid, and that it is an important and even essential ingredient of the secretion. Prof. Dalton gives the composition of the gastric juice of the dog as follows:

| | |
|---|---|
| Water, . . . . . | 975.00 |
| Organic Matter, . . . . . | 15.00 |
| Lactic acid, . . . . . . | 4.78 |
| Chloride of sodium, . . . . | 1.70 |
| " " potassium, . . . . | 1.08 |
| " " calcium, . . . . | .20 |

| | |
|---|---|
| " " ammonium, . . . . | .65 |
| Phosphate of lime, . . . . | 1.48 |
| " " magnesia, . . . . | .16 |
| " " iron, . . . . . . | .05 |
| | 1000.00 |

Besides the free acid, another most important ingredient of the fluid in question, is its organic matter, called *pepsin*, upon which depends the catalytic transformation of the food in the stomach. The action of the gastric secretion however, is confined exclusively to one class of proximate principles, the *albuminoid.* In the stomach the fatty substances are only melted by the animal heat, and such of the saccharine and starchy matters as are insoluble in water, are simply moistened and reduced to a mucilainous state; they suffer no chemical change. All albuminoid substances however, even in a solid state and entangled with other matters, are readily attacked an liquified, and whatever may have been their native characters, they are transformed into a common substance, called *albuminoid.* This substance, is held in solution by the gastric juice and is ready for absorption.

*To be Continued.*

---

